# Notes on Aceticum Acidum

**Published:** 1884-12

**Authors:** 


### NOTES ON ACETICUM ACIDUM.

A person who had been taking vinegar for some time will have a *pallid, waxen, emaciated* countenance and *vomiting;* also ulceration of the throat. A pot of vinegar on the stove is very beneficial in some diseases. You cannot cure a chronic disease if you allow your patient to use vinegar. [*Lachesis will work with it.*] When curing a chronic disease you must stop your patient from eating pickles. There are two rules I adhere to: First, in *acute* disease I generally let patients have anything they crave; second, in *chronic* disease I DO NOT. A drunkard craves that which is killing him—alcohol. Sour things must be prohibited; you must select a remedy that will antidote these tastes and cravings. For alcohol and tobacco, *Caladium* or *Asarum europeium.* Vinegar is craved by old alcoholics. Acetic acid runs as a type in many of these troubles. An old consumptive when permitted to drink vinegar will decline very rapidly, and if he is predisposed to hemorrhages, he will have them frequently.

The patient is dull, low-spirited, and depressed, both mentally and bodily. Patients addicted to the use of vinegar to excess do not recognize their own children—Acetic acid is homœopathic to this state in disease. Conditions brought on by the habitual use of coffee, tobacco, opium, and alcohol are antidoted by Acetic acid very high; nose bleed in anæmic subjects who look *pale, careworn,* and are subject to diarrhœa alternating with constipation, or have had the flow of bleeding piles suddenly checked. Under Acetic acid we have a breaking down of the red corpuscles and mucous membranes—this condition predisposes to hemorrhages. These patients take cold easily, and are subject to frequent catarrhal attacks.

*Milk impoverished, bluish, transparent, strong, sour taste* and *odor ;* different in casiene and butter (Silicia also). Patient loses flesh and gets marasmus. (Diphtheria that *begins* in the *larynx* and *goes up—Bromine.*) Acetic acid has an exudation of a fuzzy, white appearance in the throat (larynx); child had been vomiting. Wrap throat with a cloth saturated with vinegar, place a vessel containing vinegar on the stove, and give *Acetic acid high.* Croup must correspond to these symptoms: Difficult breathing from laryngeal obstruction, pseudo laryngeal croup. A croup-like, hollow sound with each INHALATION. (In the Aconite croup the sound occurs with the EXHALATION. The Aconite croup has the following: The day before has been sharp and cold; the mother has been out with the child; when she returns she puts it to bed; at about ten o'clock, or *before midnight,* the child awakens, grasps its throat, and croups. It has the appearance of ANGUISH and *fear of death.*) Acetic acid is foremost in dropsy with a diarrhœa. In diabetus insipidus there is a large amount of light-colored urine (almost as clear as water); the urinometer stands at about 1015; GREAT THIRST and EMACIATION. *Night-sweats* very profuse, and you have the same *cachetic countenance.* Night-sweats in consumptives and old chronic invalids or after typhus, when they do not recover readily. Hemorrhages from lungs, stomach, and bowels. It is useful also in stings of wasps. If you can't get *Ledum,* apply vinegar locally. *Ledum* is a grand remedy for these poisonous stings. (Whenever there is laceration and threatened tetanus—HYPERICUM.) Acetic acid is an antidote to all anæsthetic vapors. After a patient has taken Chloroform he is sick at the stomach and there is no reaction; the inhalation of diluted vinegar will restore him.

*Pale, waxen, emaciated* countenance is a very important symptom. This is Acetic acid's "red-string." In all cases where vinegar has been taken, and those cases needing it, you will find the patient *pale, waxen,* and *emaciated.* The teeth feel dull and sometimes on edge. An important symptom is, indications of white film *low down* in the *fauces.* This is a remedy for *croup,* some *dropsies,* and diabetus insipidus—not where there is *sugar,* but a great quantity of watery urine. There is an inflammation of the throat which is cured by wrapping it with a flannel saturated with vinegar. Children swallow with some difficulty even a teaspoonful of cold water. In connection with this case the child will be *pale, waxen,* and *emaciated.* The patient is *run down* in this remedy. Ashy colored exudations in the throat. An important symptom is: *No thirst* in *fever,* but there *is thirst* in

*dropsy.* You would hardly think of any other remedy in consumption with dropsy of the feet and legs extending to the knees, especially if there is *great thirst. No thirst* in *croup* or *fever.* It also disturbs very greatly the pneumogastric nerve; hence we have nausea and vomiting. It may go on to ulceration of the stomach, and with this symptom we have a *pale, waxen* countenance. The stomach becomes so debilitated that the patient vomits after every particle of food, but it is said he retains water. (The Bismuth patient vomits every drop of water as soon as it touches the stomach.) (In *Arsenicum* the water when taken is either vomited or *passes* THROUGH the patient immediately.) (Under PHOS. the water comes up as soon as *warm* in the stomach.) Vomiting after every meal. The contents of the stomach feel as if in a ferment. Cold sweat on the forehead. Diarrhœa with swelling of the limbs and feet (dropsy) in phthisis. Diarrhœa in the latter stages of typhus and typhoid fever. Now, in this *latter* diarrhœa there is *no thirst,* but in the *former* diarrhœa there is *violent thirst.* In profuse hemorrhoidal bleeding you would not think of this remedy if the patient was *stout* and *robust* and *hearty,* but if *waxen, careworn, tired,* and *broken down.* A guiding symptom in pregnancy is: Sour belching and vomiting, with profuse water-brash and salivation, day and night. A person can be salivated as well by vinegar as by *Mercury. Calcarea* is very similar to this state during pregnancy. (A remedy for vomiting in pregnancy is *Symphoricarpus racemosus.*) Local applications of vinegar for hemorrhages after labor.

